# Fe(III) Mixed IP6@Au NPs with Enhanced SERS Activity for Detection of 4-ATP

**DOI:** 10.1038/s41598-020-62495-w

**Published:** 2020-04-01

**Authors:** Lei Zhang, Yi-jin Weng, Xiao Liu, Wen Gu, Xia Zhang, Lei Han

**Affiliations:** 1 School of Materials Engineering, Shanghai University of Engineering Science, Shanghai, 201620 China; 2 College of Chemistry and Pharmaceutical Sciences, Qingdao Agricultural University, Qingdao, 266109 China

**Keywords:** Environmental sciences, Nanoscience and technology

## Abstract

Surface Enhanced Raman Scattering (SERS) has been widely applied in many research fields such as biological detection and chemical analysis. However, for the common Au nanoparticles, it’s too hard to guarante the three aspects: the great enhanced effect, the controllable aggregation and the uniformity of nanoparticles, the environmental friendliness and biocompatibility of nanoparticles. In this paper, phytate acid (IP6)-coated Au nanoparticles (IP6@Au NPs) are more stable and have a higher enhancement factor than Au nanoparticles. In order to achieve the uniformity of the spherical IP6-coated@Au nanoparticles (IP6@Au NPs), IP6 was used as a soft template. In the presence of IP6, IP6@Ag nanoparticles were first synthesized by reducing AgNO_3_ with trisodium citrate, then IP6@Au NPs were obtained by reducing HAuCl_4_ with Ag nanoparticles. The IP6@Au NPs exhibit excellent Raman signal enhancement by using p-aminothiophenol (4-ATP) as the probe molecules. The effects of Fe^3+^ on the performance of IP6@Au NPs SERS substrates were also studied. The results show that SERS has the best enhancement effect when adding proper amount of Fe^3+^ (0.56 PPM), and the limit of detection was 10^−7^ M 4-ATP.

## Introduction

Surface Enhanced Raman Scattering (SERS) has become a spectral analysis measurement technique which utilizes nanoscale rough surfaces and metal nanoparticles as the magnified detection substrates. Due to its higher resolution, sensitivity and detection capability, SERS has attracted an increasing number of researchers, which can be used to characterize minute quantity of molecular vibration information^1,2^ and overcomes the shortcomings of traditional Raman spectroscopy. At present, most of the nanomaterials are for directly detecting the presence of toxic substances, or indirectly detecting other substances through surface modification and adsorption probe molecules. For example, the dyes and dye intermediates^3^ of rhodamine (RhB) or p-aminothiophenol (4-ATP).

Regarding the enhancement mechanism of SERS, there is still no consensus in the academic world^4^. Most scholars believe that SERS enhancement mainly consists of physical enhancement and chemical enhancement. A large number of experimental studies have shown that the influencing factors of the enhancement process are very complex. In many systems, these two factors may in coordination with each other at the same time. The physical enhancement is mainly to induce a local electromagnetic field through the rough metal surfaces in the energy excitation state, which generating “hot spots” and improving the SERS enhancement effect^5^. Therefore, the optimization of physical enhancement mechanism has become the main way to improve the SERS enhancement factor in the context of the little research on chemical enhancement. Previously, the SERS-active substrates were fabricated by electrochemically roughening the surfaces of the electrode metals^6^, by sputtering the metal target to form the metal island films^7^, and so on. Metal colloids were later found to be prepared easily and possess higher enhancement factors, especially for colloids of Ag and Au^8,9^. The size control of the colloids, the aggregation degree or aging degree of the colloids have become two major problems. The SERS substrates we prepared can be combined well with many probe molecules while reducing the aggregation degree of nanoparticles, thus ensuring the reproducibility of the SERS signal and avoiding complex spectral interferences or cluster surface deactivations^10^.

4-ATP is a highly toxic chemical which is often used as pesticide, pharmaceutical, or dye intermediate. It denatures proteins and is absorbed by the body. Meanwhile, it forms methemoglobin and causes purpura. Due to its nature, although the peaks of 4-ATP can be detected, the plasmon resonance generated in the energy excited state can convert 4-ATP into 4-NTP easily, which makes the detection difficult and the spectral interpretation complicated. Therefore, the interpretation of Raman characteristic peaks of 4-ATP has been controversial, which not only affects the direct detection of 4-ATP, but also affects its application in indirect detection of proteins^11^.

Currently many biocompatible based SERS-active substrates are reported for single molecule detection, and their excellent enhancement performance has realized the single molecule detection of many substances^12,13^. In this paper, phytic acid coated Au nanoparticles (IP6@Au NPs) are used as the substrates, the energy transfer caused by plasmon resonance is reduced and 4-ATP conversion is effectively avoided. In addition, the addition of Fe^3+^ can increase the number of “hot spots” formed by the combination with phytic acid (IP6), which improves the stability and enhance the effects of SERS substrates. It also lays a scientific foundation for the indirect detection of Fe^3+^ in water.

## Experiment

### Chemicals and materials

All chemicals were of analytical grade or the highest purity among the currently available commercial markets. Ferric chloride hexahydrate (FeCl_3_•6H_2_O, yellow block solid), chloroauric acid (HAuCl_4_), silver nitrate (AgNO_3_), phytic acid (IP6, inositol hexaphosphate) and trisodium citrate (Na_3_C_6_H_5_O_7_•2H_2_O) were purchased from Sinopharm Chemical Reagent Co., Ltd. (Shanghai, China). Para-aminothiophenol (4-ATP) was purchased from Aladdin Reagent Co., Ltd. (Shanghai, China). The Fe^3+^ solution was prepared by adding FeCl_3_•6H_2_O to deionized water as a stock solution. Milli-Q water (18.2 MΩ • cm) was used throughout the experiments, and all glasswares were cleaned with in aqua regia before used.

### Experimental methods

#### Preparation of IP6@Ag NPs

**Figure 1:**
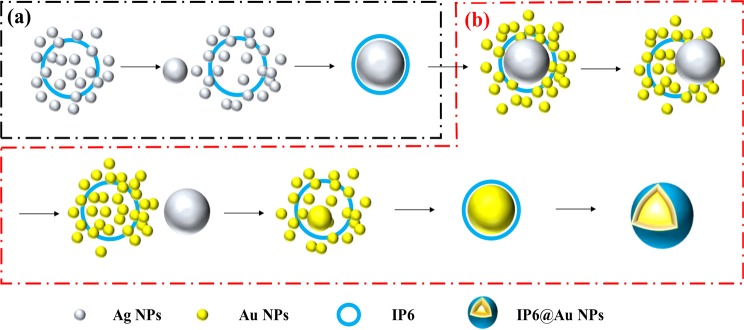
The principle diagram of two-step preparation of IP6@Au NPs (**a**) The schematic diagram of synthetic IP6@Ag NPs; (**b**) The schematic diagram of synthetic IP6@Au NPs.

The Ag nanoparticles were prepared by a method which is similar to Lee’s except for the presence of IP6. IP6@Ag NPs were prepared by reducing silver nitrate with trisodium citrate. The detailed steps were as followed: Dissolve 0.0255 g of AgNO_3_ in 150 mL of deionized water. Add 5 mL of 1 mM IP6 solution to the above solution, place the solution in a round bottom flask and heat it rapidly to boiling. After the solution was heated to boiling, 3 mL of 1% trisodium citrate solution was added slowly under vigorous stirring^14^. The reaction of the solution kept 6 h under boiling temperature to obtain Ag colloids. An appropriate amount of Milli-Q water was added to obtain 125 mL of Ag colloids (the final concentration of Ag was 1.2 mM). The principle is shown in Fig. 1a.

#### Preparation of IP6@Au NPs

In this paper, IP6-coated Au NPs were obtained by using IP6-coated Ag NPs to reduce HAuCl_4_. The specific steps were as followed: first, took 10 mL of the prepared IP6@Ag NPs diluted to 25 mL with appropriate amount of deionized water. Then added 5 mL of 1 mM HAuCl_4_ slowly during stirring of the mixtures at the meantime. The solution quickly turned white, then turned into cyan, and finally changed into rose red slowly. IP6@Au NPs were obtained by heating the mixtures at 50 °C for 30 min. In addition, the sample of the nanoparticle colloids (IP6@Ag@Au NPs) was taken from intermediate process (about 15 minutes after the start of the reaction) for subsequent test comparison. The principle is shown in Fig. 1b^15^.

### Preparation of SERS Substrates and detection of 4-ATP

2 mL of prepared IP6@Au NPs were taken out, and 1 mL of different concentrations (0 PPM, 0.28 PPM, 0.56 PPM, 0.84 PPM, 1.12 PPM) of Fe^3+^ solutions was added to form IP6@Au NPs@Fe^3+^. After the above mixed solution was stirred at room temperature for 1 min on a thermostatic magnetic stirrer, 1 mL of the probe molecules 4-ATP were added, and then stirred continually for 1 min to form IP6@Au NPs@Fe^3+^@4-ATP. The prepared SERS substrates were then centrifuged for 30 minutes at 10400 r/min. Since the different concentrations of 4-ATP were added during the experiments, the centrifugation time will be different, as long as the precipitation occurs during the centrifugation. Subsequently, 10 μL of the precipitate was taken out, dropped on a concave glass slide, dried and characterized by a HORIBA’s focused Raman system (Model: HR Evolution), using 532 nm laser, and the Raman signal was detected at the laser of 1% ND Filter.

## Results and Discussion

### SEM images and UV-Vis absorption spectroscopy

**Figure 2:**
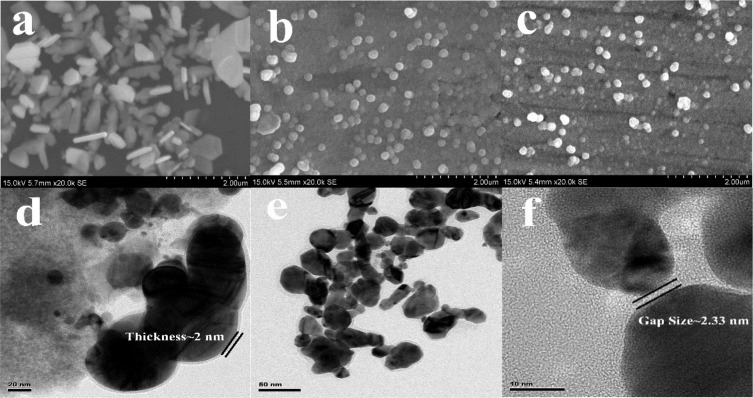
SEM images of the nanoparticles (**a**) IP6@Ag NPs; (**b**) IP6@Ag@Au NPs; (**c**) IP6@Au NPs and TEM image of the nanoparticles (**d**) IP6@Au NPs; (**e**) IP6@Au NPs@Fe^3+^; (**f**) IP6@Au NPs@Fe^3+^ with higher magnification.

The morphology of the IP6 micelle-coated metal nanoparticles synthesized by the above method was characterized by Scanning Electron Microscopy (SEM) and Transmission Electron Microscopy (TEM). Fig. 2a is the SEM image of IP6@Ag NPs. Due to the different addition amount and rate of trisodium citrate, the nanoparticles exhibit anisotropy and grow into snowflakes along different crystal faces^16–19^. Fig. 2b shows the nanoparticles image during the reduction reaction of IP6@Ag NPs with HAuCl_4_. When the reduction reaction was completely over, the nanoparticles of IP6@Au NPs exhibit a uniform spherical state as shown in Fig. 2c. Fig. 2d shows the TEM image of IP6@Au NPs, and clearly indicated that IP6 (~2 nm) is uniformly wrapped on the surface of Au nanoparticles. The TEM image of IP6@Au NPs after adding Fe^3+^ is shown in Fig. 2e. Due to the chelation of Fe^3+^ and IP6, the distance between the nanoparticles is effectively regulated. As shown in Fig. 2f, the distance between the nanoparticles (Gap size ~ 2.33 nm) is more likely to generate an electromagnetic field, thereby forming a large number of “hot spots” and improving the SERS enhancement performance.

**Figure 3:**
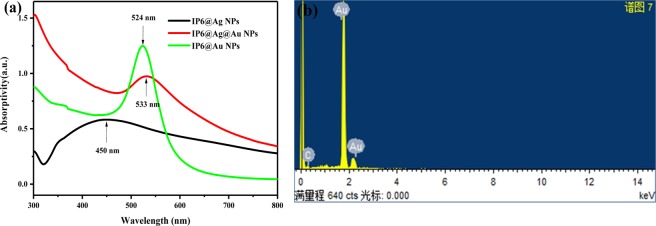
(**a**) The UV-Vis absorption spectra of IP6@Ag NPs, IP6@Ag@Au NPs and IP6@Au NPs (**b**) The EDS image of IP6@Au NPs.

In order to study the formation process of nanoparticles, we carried out UV-visible absorption spectroscopy tests on nanoparticles at different stages, and estimated the composition of the nanoparticles by absorption peaks. Fig. 3 shows the UV-Vis absorption spectra of IP6@Ag NPs and IP6@Au NPs and the solution (IP6@Ag@Au NPs) during the replacement process. In Fig. 3a, the highest absorption peak of Ag NPs is around 450 nm, and the absorption spectrum is broadened due to the influence of IP6 micelles. During the replacement process, the simultaneous presence of Ag NPs and Au NPs, the overlap of two absorption peaks formed, the absorption spectrum becomes high at around 450 nm, and a significant absorption peak of Au NPs has been formed at 533 nm. Finally, the absorption peak at 450 nm decreases significantly, and the absorption peak at 524 nm increases, indicating that the replacement reaction was completed, and the formation of IP6@Au NPs with uniform sizes and favorable dispersion. It can also be seen from the EDS image that the replacement reaction was completed, and the obtained IP6@Au NPs does not contain Ag. According to the literature, Ag NPs usually has a strong plasma absorption peak around 400 nm^15,20,21^. With the increase of Ag NPs, the absorption spectrum broadens and shifts, which is in agreement with the experimental results. When studying the scattering of metal colloidal particles, Mie scattering is established^22^, and the Mie’s rigorous solution to light scattering is obtained by the Maxwell’s equation of electromagnetic waves, which solves the scattering law of uniform particles of arbitrary diameter and arbitrary composition^22^. This rule also demonstrates the results of the UV-Vis absorption spectra.

**Figure 4:**
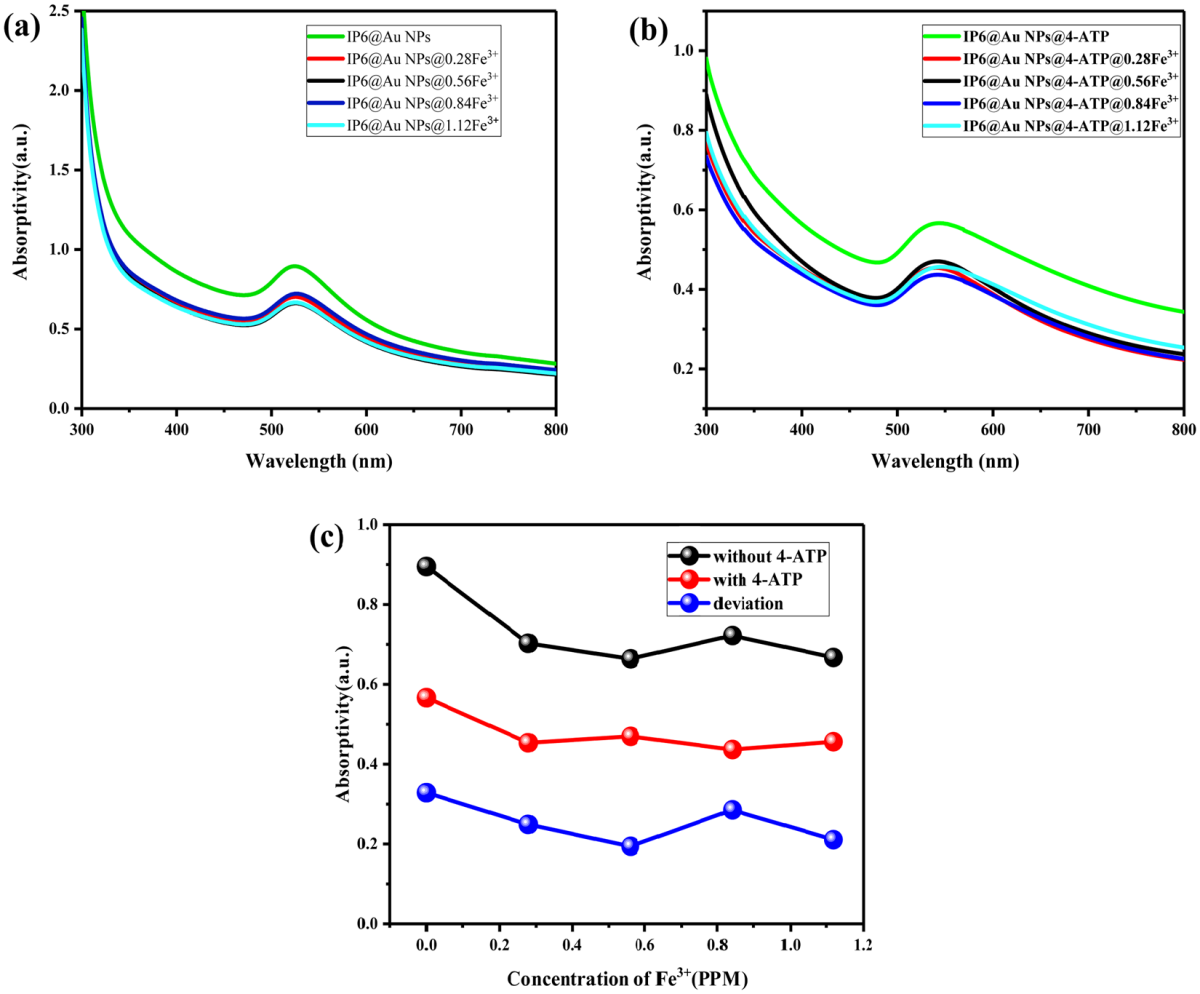
UV-Vis absorption spectra of (**a**) IP6@Au NPs@Fe^3+^, (**b**) IP6@Au NPs@4-ATP @ Fe^3+^, (**c**) Deviation of the highest absorption peak without and with 4-ATP as the probe molecules. Among them, the concentration of Fe^3+^ is 0 PPM, 0.28 PPM, 0.56 PPM, 0.84 PPM, 1.12 PPM.

In Fig. 4a, with the addition of Fe^3+^, the absorption peak of IP6@Au NPs at 520 nm becomes gentle. The researchers^23^ mentioned that as the reaction time prolongs, the nanoparticles will continue to grow and the absorption spectrum will shift, and this phenomenon also can be supported by the Mie theory^22^. It can be seen in Fig. 4a that when the concentration of Fe^3+^ increases, the curves of the UV-Vis absorption spectra first decreases to the lowest at 0.56 PPM, and then rises with 0.84 PPM, 1.12 PPM of Fe^3+^, which is attributed to the aggregation of nanoparticles caused by the formation of Fe-O between IP6@Au NPs^21^.

When the probe molecule 4-ATP is added (Fig. 4b), the overall intensity of the UV-Vis absorption spectra is weakened and a slight red shift occurs (~20 nm). From the aspect of intensity, it is worth noticed that the UV-Vis absorption spectra of the SERS substrates added with Fe^3+^ has the opposite result of Fig. 4a except for the decrease in intensity. The lower the absorption spectra intensity in Fig. 4a, the smaller decrease in the intensity of the ultraviolet-visible absorption spectra after the addition of 4-ATP (Fig. 4b), and the deviation is shown in Fig. 4c. The magnitude of the difference indicates the change caused by the addition of the probe molecule 4-ATP, and the smaller in intensity decrease, the more stable of the substrates, the better enhancement of the SERS substrates^23,24^. For this phenomenon, we believe that the Fe-O formed after the mixing of Fe^3+^, Fe-O linkage in IP6@Au NPs@Fe^3+^ NPs makes the solution more stable. From the aspect of the absorption band, since the solvent of the 4-ATP solution is anhydrous ethanol, the solvent effect came into being. A hydrogen bond may be formed between the solvent molecule and the solute molecule, or the dipole of the polar solvent molecule increases the polarity of the solute molecule, causing a change in the energy level of the solute molecule, thereby causing migration of the absorption band (red shift)^25,26^.

### Enhancement mechanism of IP6@Au NPs@Fe^3+^ as SERS substrates

**Figure 5:**
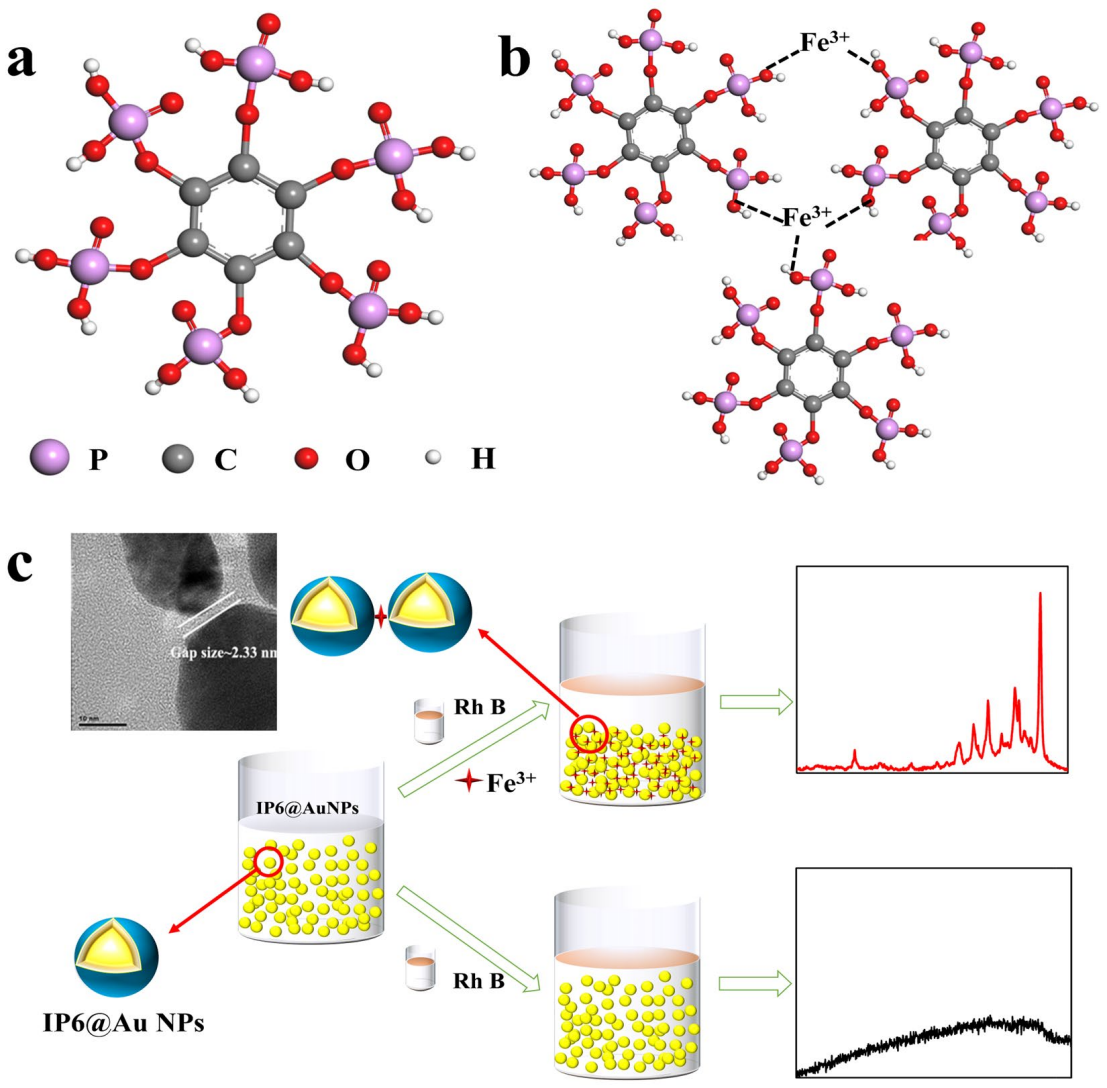
(**a**) The structure of inositol hexaphosphate (IP6). (**b**) The interaction between Fe^3+^ and IP6@Ag NPs. (**c**) Schematic diagram of IP6@Au NPs as a SERS substrate by adding Fe^3+^ to increase “hot spots”.

IP6 is an environmentally friendly, readily available natural compound. It is composed of inositol and six phosphate ions (Fig. 5a), and is often used as a control agent, chelating agent and stabilizer in a wide range of industries such as food and medicine^27^. Because of its structure containing six phosphates separately on both sides of the cyclohexane, IP6 and its salts are liable to chelate with metal ions. As shown in Fig. 5b, when an appropriate amount of Fe^3+^ is added, Fe^3+^ coordinate with oxygen atoms in IP6 chelate to form Fe-O^20,21^. The formation of Fe-O linkage leads to a controllable aggregation of Au NPs, brings the closer distance between Au NPs, which produce plenty of “hot spots” existing in the nanoscale junctions in metal nanostructures. Meanwhile, the aggregated IP6@Au NPs could produce the large electromagnetic field, which improves the enhancement performance of SERS substrates. The principle is shown in Fig. 5c.

### Application of IP6@Au NPs@Fe^3+^ in detection of 4-ATP

So-called “hot spots”, on the metal nanostructures due to the excitation of localized surface plasmon resonance (LSPR)^5,28^. When the SERS substrates stimulated by energy, the electron transfer increases sharply, which leads to increase the “hot spots” in the nanoscale junctions in metal nanostructures, thus achieving the effect of SERS enhancement. It also can be seen in the TEM image of IP6@Au NPs with 0.56 PPM Fe^3+^ (Fig. 2e) that the nanoparticles start to aggregate in a wide range, and the distance between the nanoparticles shrinks (~2.33 nm) to form a large number of “hot spots” (Fig. 2f). So we suspect that as the concentration of Fe^3+^ increases, the number of “hot spots” increases and then decreases, and the SERS enhancement effect also increases and then decreases.

**Figure 6:**
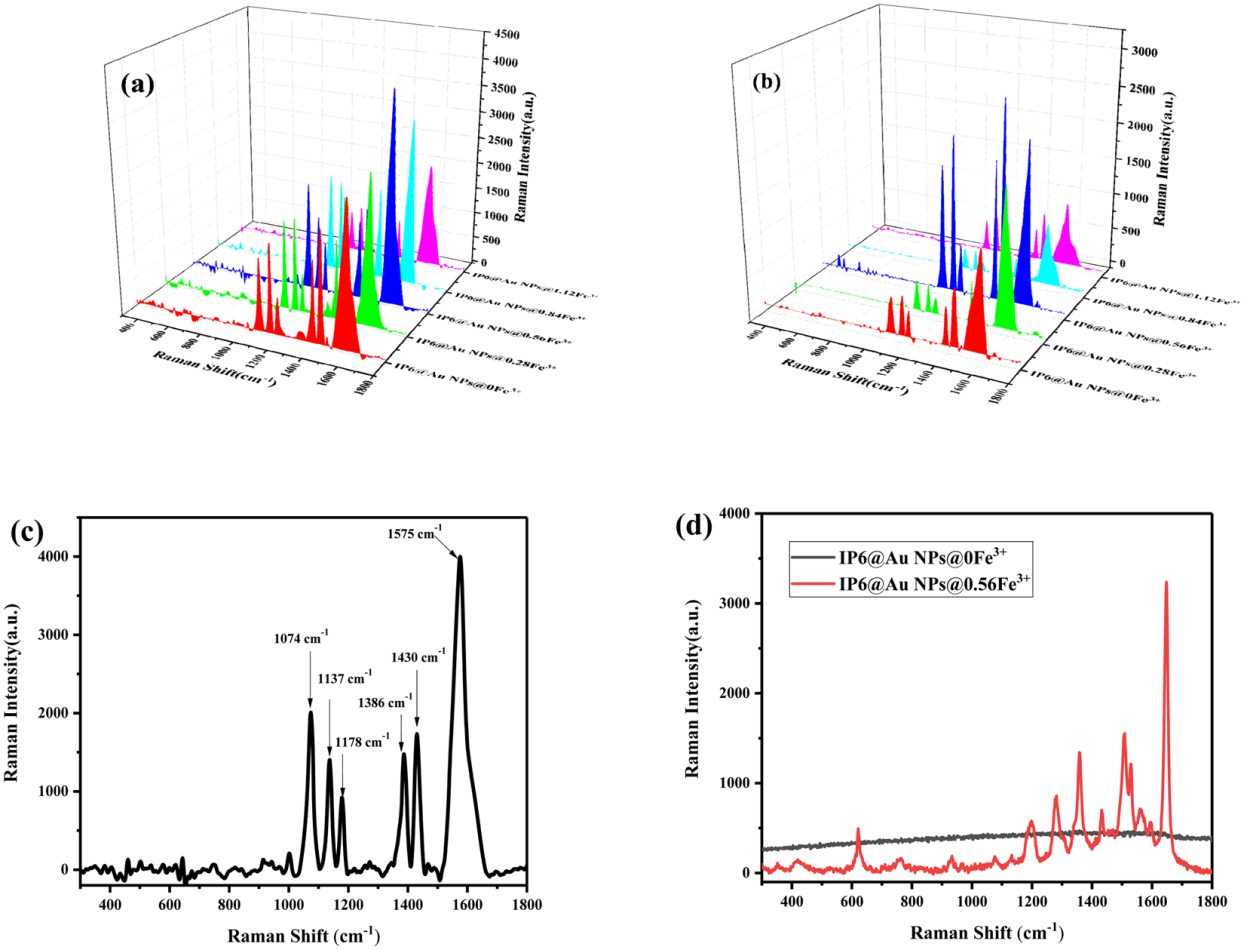
The Raman spectra of 4-ATP (**a**) 10^−3^ M, (**b**)10^−5^ M on IP6@Au NPs@ Fe^3+^ at the addition of different Fe^3+^ (0 PPM, 0.28 PPM, 0.56 PPM, 0.84 PPM, 1.12 PPM), (**c**) The Raman spectra of 4-ATP (10^−3^ M) on IP 6@AgNPs with 0.56 PPM Fe^3+^, (**d**) The Raman spectra of RhB (10^−7^ M) on IP 6@AgNPs without Fe^3+^ and with 0.56 PPM Fe^3+^.

Fig. 6a,b are the SERS spectra of 4-ATP (10^−3^ M, 10^−5^ M) on IP6@Au NPs with different concentrations of Fe^3+^ (0~0.84 PPM). When the concentration of Fe^3+^ increases, the Raman enhancement effect enhanced and then weakened, the best enhancement effect was 0.56 PPM Fe^3+^. This was in agreement with the UV-Vis absorption spectra of Fig. 4a, which also validates our conjecture in the most stable formation of Fe-O linkage. When the Fe^3+^ concentration was 1.12 PPM, the UV-Vis absorption spectra broadening and the intensity is weakened due to particle agglomeration (Fig. 4a), which was almost the same as 0.56 PPM, but the Raman signal was not enhanced. It may be the presence of high concentration of Fe^3+^ and this made a large amount of IP6@Au NPs agglomerate. Because the particles formed by agglomeration were too large, there is no effective “hot spots” formation, which leads to the phenomenon of Fe^3+^ overloading, so the Raman signal is suppressed directly in the aforementioned SERS experiment. In addition, Fig. 6d shows the Raman spectrum of RhB. The results show that the SERS substrate with 0.56 PPM Fe^3+^ can increase the Raman signal by several orders of magnitude and without interference of other samples.

In order to see the Raman characteristic peak of 4-ATP more clearly and intuitively, Fig. 6c shows the characteristic peak of 4-ATP (10^−3^ M) at 0.56 PPM Fe^3+^. It could be seen from the literature, there were many explanations for the Raman characteristic peak of 4-ATP. In the early report of Osawa group^29^, the peaks appearing at 1137 cm^−1^, 1386 cm^−1^, and 1430 cm^−1^ were “b2” vibration modes, the peak vibration modes of 1074 cm^−1^, 1178 cm^−1^, and 1575 cm^−1^ were attributed to the “a1” symmetry. Later, in the article of Zhao^30^, they systematically calculated and verified the vibration attribution of 4-ATP. It is considered that the 4-ATP molecule can be approximated as having C_2ν_ symmetry. DMAB belongs to the C_2h_ symmetry molecule, and its main Raman peaks (1137 cm^−1^, 1386 cm^−1^, 1430 cm^−1^) belonged to the “ag” vibration mode, and other strong Raman peaks belonged to the “a1” vibration mode. In the latest report^31^, the peak vibration modes of 1074 cm^−1^, 1178 cm^−1^, and 1575 cm^−1^ were attributed to v_C-C_ + v_C-S_, β _C-H_ and v_C-C_. The peaks of DMAB emerged in the SERS spectra at 1137 cm^−1^, 1386 cm^−1^, 1430 cm^−1^ assigned to its β_C-H_ + v_C-N_, v_N-N_ + v_C-N_ and v_N-N_ + β_C-H_.

**Figure 7:**
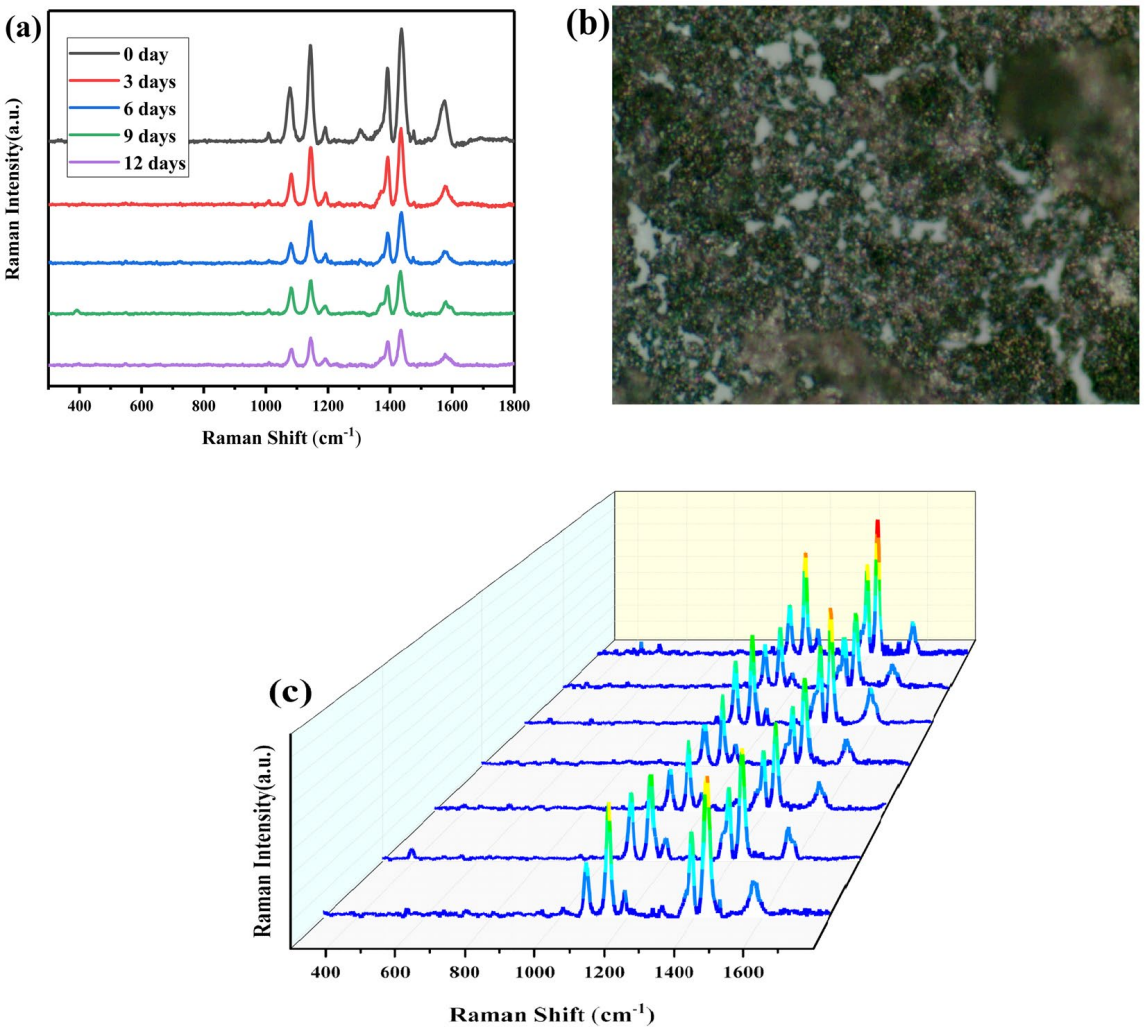
(**a**) Raman spectra of the substrate after being left at different times (**b**) Raman imaging with a 50× objective (**c**) Raman spectra of seven different positions of the same sample (in Fig. 7b).

In order to verify the reproducibility and stability of the substrate, we obtained the corresponding SERS signal of stability experiments and repeatability experiments. The results are shown in Fig. 7, where Fig. 7a shows the Raman spectra of the same substrate after being left at different times, and Fig. 7c shows the Raman spectra of seven different positions of the same sample (the image of the substrate shows in Fig. 7b). It can be seen that the reproducibility and stability of IP6@Au NPs are excellent.

**Figure 8:**
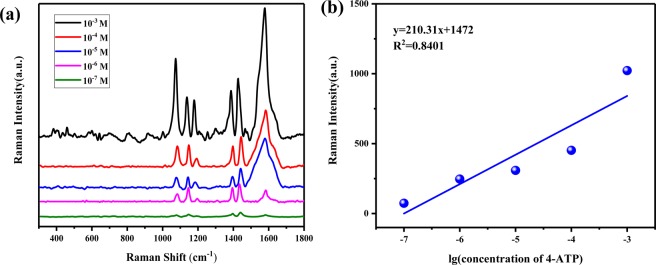
(**a**) Raman spectra of different concentrations of 4-ATP (10^−3^M, 10^−4^ M, 10^−5^ M, 10^−6^ M, 10^−7^ M) at the concentration of 0.56 PPM Fe^3+^. (**b**) A line graph of Raman peak intensity at 1386 cm^−1^and 4-ATP concentration.

Based on previous phenomenon, we obtained the Raman signals of different concentrations of 4-ATP at the Fe^3+^ concentration of 0.56 PPM, as shown in Fig. 8a. When the SERS system was used to detect 4-ATP down to the trace level of 10^−7^ M, the Raman peak could still be observed clearly. From this we can also see that the reproducibility of IP6@Au NPs@Fe^3+^ for Raman enhancement is favorable. Then the Raman spectra at 1386 cm^−1^ of different concentrations of 4-ATP was processed, as shown in Fig. 8b. It could be seen that the intensity of the Raman peak has a good linear relationship with the concentration of 4-ATP, and the concentration of 4-ATP could be assessed by the intensity of the Raman peak. In addition, the detection limits of 4-ATP were compared with other nanomaterials, and the results are shown in Table 1.

**Table 1:** Detection limits of 4-ATP for different nanomaterials.

Nanomaterials	Limits of detection	References
SiO_2_@Ag@SiO_2_ NPs	10^−4^ M	^32^
gold-silver framework monolayer	10^−4^ M	^33^
Silver nanoparticle-doped diamond-like carbon film	10^−5^ M	^34^
ZnO-Au	10^−5^ M	^35^
Fe_3_O_4_@SiO_2_-SO_3_H@PPy@Au spheres	10^−6^ M	^36^
Ag/mSiO_2_	10^−7^ M	^37^
ZnO/Ag	10^−7^ M	^38^

## Conclusion

In this paper, IP6@Au NPs were prepared by redox method. Fe^3+^ and IP6@Au NPs were mixed in proportion to obtain a composite SERS substrate IP6@Au NPs@Fe^3+^ with excellent performance. 4-ATP was used as a probe molecule for Raman spectroscopy. By characterizing the enhanced substrate with different concentrations of Fe^3+^, it was found that with the increase of Fe^3+^ concentration, the enhancement effect increased and then decreased. It was finally determined that it had a very excellent reinforcing effect under the condition of low concentration of Fe^3+^ (0.56 PPM), and the detection limit of 4-ATP can reach at 10^−7^ M. When higher concentrations of Fe^3+^ (0.84 PPM and 1.12 PPM) were incorporated, Fe^3+^ caused overload of the SERS substrate, which makes the nanoparticles agglomerate seriously and failure to form effective “hot spots” and affects the test signal of the original probe molecule, resulting in a weakened SERS enhancement effect. In addition, the concentration range of 4-ATP could be effectively obtained by linear fitting of the 4-ATP Raman peak.
